# Game of Hearts

**DOI:** 10.5334/jbr-btr.1040

**Published:** 2016-02-18

**Authors:** Katrien Gieraerts, Naïm Jerjir

**Affiliations:** 1 UZ Leuven, BE

In this quiz-based presentation we aimed to give an overview of basic cardioradiological anatomy, anatomical variations and pathologies. Featured topics include the normal anatomy of the aortic valve and its variations, some normal structures and their pitfalls, anomalies in the coronary arteries, and finally, selected congenital (scimitar vein, tetralogy of Fallot) and acquired (myocarditis, amyloidosis, ventricular aneurysm after myocardial infarction, Takotsubo, noncompaction) cardiac pathologies. Imaging modalities include conventional radiography, computed tomography, and magnetic resonance imaging. Teaching points were provided.

## Competing Interests

The authors declare that they have no competing interests.

